# Are Two Interviews Better Than One? Eyewitness Memory across Repeated Cognitive Interviews

**DOI:** 10.1371/journal.pone.0076305

**Published:** 2013-10-03

**Authors:** Geralda Odinot, Amina Memon, David La Rooy, Ailsa Millen

**Affiliations:** 1 Research and Documentation Centre, Ministry of Security and Justice, The Hague, The Netherlands; 2 Royal Holloway, University of London, London, United Kingdom; 3 School of Psychology, University of Abertey Dundee, Dundee, United Kingdom; 4 School of Psychology, University of Portsmouth, Portsmouth, United Kingdom; University of Akron, United States of America

## Abstract

Eyewitnesses to a filmed event were interviewed twice using a Cognitive Interview to examine the effects of variations in delay between the repeated interviews (immediately & 2 days; immediately & 7 days; 7 & 9 days) and the identity of the interviewers (same or different across the two repeated interviews). Hypermnesia (an increase in total amount of information recalled in the repeated interview) occurred without any decrease in the overall accuracy. Reminiscence (the recall of new information in the repeated interview) was also found in all conditions but was least apparent in the longest delay condition, and came with little cost to the overall accuracy of information gathered. The number of errors, increased across the interviews, but the relative accuracy of participants’ responses was unaffected. However, when accuracy was calculated based on all unique details provided across both interviews and compared to the accuracy of recall in just the first interview it was found to be slightly lower. The identity of the interviewer (whether the same or different across interviews) had no effects on the number of correct details. There was an increase in recall of new details with little cost to the overall accuracy of information gathered. Importantly, these results suggest that witnesses are unlikely to report everything they remember during a single Cognitive Interview, however exhaustive, and a second opportunity to recall information about the events in question may provide investigators with additional information.

## Introduction

Police and Courts rely heavily on the accuracy of information gathered from eyewitnesses. It is not unusual for witnesses to be interviewed more than once over subsequent sessions. The effects of repeated interviews are a source of controversy in legal contexts and the literature has highlighted advantages and disadvantages [1–3]. Repeated recall of the same event is associated with confidence inflation [4,5], retrieval induced forgetting [6], and the increased likelihood of misinformation effects, especially when the rehearsal is selective or biased [7,8]. There are also possible benefits for witnesses who are given more than a single opportunity to recall their experiences [2,9–14]. Research on repeated interviewing is, however, surprisingly scarce. While the number of ‘single interview’ eyewitness studies are overwhelming. To our knowledge there are no published studies in which adult witnesses have been interviewed repeatedly with a research based protocol about a rich, ecologically valid, experience (see also 16). The main goal of the current study is to provide novel insight into the costs and benefits of repeated interviewing.

Research has shown that people can retrieve new details at later recall attempts which they did not retrieve in an earlier attempt (i.e., reminiscence) and some studies have reported an increase in the total amount of information that was reported in a subsequent recall compared to the initial recall (i.e., hypermnesia, see 17 for a review). Reminiscence has been consistently reported in studies of children’s eyewitness memory. For example, La Rooy, Pipe, & Murray [14] examined the effects of a short (immediate, 24 hrs) and long delays (6 months) on reminiscence and hypermnesia. They reported hypermnesia effects but only in interviews that occurred immediately and again 24 hours after an event (Experiment 1). Recall of new items was seen in both the shorter and longer delays examined, but fewer new items were recalled after a 6-month delay between interviews. In a systematic field study of children who had allegedly been sexually abused, Hershkowitz & Terner [12] examined the records of repeated interviews and noted that almost 25% of the information given by the witnesses during the second interview comprised new, previously unreported, details (reminiscence). Based on their findings the researchers concluded that repeated forensic interviews can elicit new information and preserve central details. Moreover, repeated interviews may facilitate recall of new details while preserving previously recalled details. It is important to test this hypothesis because it challenges the assumption among legal professionals that consistency is an indicator of accuracy (see [1,])

Obtaining full and accurate witness accounts is critical and various interview protocols have been used over the years to achieve high quality testimonies (see [19]; for a recent review). The Cognitive Interview (CI) is probably among the best-known technique for use with adults [20]. The CI has been shown to increase the amount of information that can be obtained from an eyewitness in numerous laboratory studies. However, the effectiveness of the CI across subsequent interviews in combination with the delay between the event and first interview or the delay between two interviews has never been studied before [16]. Only three studies have examined the effect of repeated recall using the CI in the initial and subsequent interview [22]. These studies had delays ranging from 5 min to 2 days for the first interview and 10 to 14 days for the second. The overall finding in each study was a gain in correct details in the first interview when a CI was used as compared to a control condition. However, none of the studies reported apparent advantages of two subsequent Cognitive Interviews at Time 1 and Time 2 and no carryover effects were reported. One critical variable was omitted from this earlier research. The eyewitness literature indicates that the length of delays between first and subsequent interviews can have varying effects on the accuracy of eyewitness memory [24]. Increases in the reporting of information as a result of repeated interviewing (hypermnesia) are more likely when there are short delays between memory tests [,,25] and the magnitude of hypermnesia decreases with longer delays between the event and the repeated memory tests [17]. Furthermore, reminiscence was consistently found irrespective of whether or not hypermnesia occurred. In other words, it is not unusual for people to report new details across repeated interviews, and therefore, understanding the dynamics of repeated interviewing is highly relevant during criminal investigations where new information can be crucial.

In the present study, we examine the effects of repeated interviewing using three different combinations of interview delay. The interview delays were selected on the basis that our memory is likely to be subject to most forgetting immediately after the event in question and then ‘levels off’ over time [28]. However, an early test can also offset forgetting [,21], and previous memory retrieval can also increase the likelihood that the same information is recalled again [6,,30]. The effects of repeated interviews need therefore be understood in the context of varying recall delays.

While there may be benefits to repeated interviewing, there is also a concern that it may contribute to a decrease in witness credibility due to inconsistencies in details reported across different interviews. Judges and legal professionals are very suspicious of changes in testimony that are assumed to be indicative of flaws in memory (an unreliable account) or dishonesty [1,,33]. Contrary to this belief research has shown that accuracy and consistency are not strongly related so the perception that an inconsistent witness is unreliable is misleading [1,]. In order to clarify the relationship between consistency and accuracy, in this research recall of details across interviews is examined on an item-by-item basis. This will provide precise information about the quality of details elicited with repeated interviews.

In legal contexts, eyewitnesses may be interviewed for different purposes by a variety of individuals including police officers, social workers, lawyers, judges and psychologists. The average number of subsequent interviews for child witnesses was found to be 4 times [3]. Having to talk to a different individual each time, particularly about a distressing experience, can cause discomfort for witnesses as well as negatively impacting the outcome of an interview. However, having the same interviewer on subsequent sessions should reduce the social demands for consistency and increase the expectation that new information should be provided. There is evidence from just one previous study that the social context of the interview may vary with interviewer identity and effect recall. In a study by Bjorklund, et al. [36], children and adults responded to correct or misleading questions asked by either the same or a different interviewer across recall sessions. Counter to their hypothesis, error rates were higher when participants were interviewed by a different person to that who had questioned them earlier. Therefore, this highly relevant topic for practitioners needs more attention from researchers. For that reason, in the current study the two interviewers conducted the interviews with the interviewers fully counterbalanced across the interviews to avoid confounds. This also allowed us to examine possible interviewer effects due to whether the same of a different interviewer conducted the interviews. Given the findings of Bjorklund et al. [36], we also included the factor of interviewer familiarity or unfamiliarity in the analysis given its potential relevance for applied settings. We hypothesized that when the same interviewer was used, the social demand for consistency across retellings would be reduced and witnesses would focus on providing new details. Interviewer effects have not previously been examined in relation to the CI.

To sum up, based on the above review, there may be benefits for witnesses who are given more than one opportunity to report their memories for an event, namely that they have the opportunity to provide new information. This however, has never been tested before with adults witnesses recalling repeatedly a rich episodic memory. The first test of this hypothesis where retention interval and interviewer are manipulated as variables and a high quality interview procedure (the Cognitive Interview) is used. Based on previous findings, it was predicted that reminiscence and hypermnesia would be found with repeated interviewing. In addition, we expected a decrease in the amount and accuracy of new items (reminiscence) when there is a delay between interviews, and when there are delays before the repeated interviews. To explore the effects of delay we therefore chose an ecologically valid interval of one week between our interviews. We also hypothesized that consistency would not be related to overall accuracy of memory. However, we were also particularly interested in providing new knowledge by placing these phenomena in a wholly applied context.

## Methods

### Participants

A group of 80 undergraduate students and employees of the university (44 female and 36 male, age *M* = 22.59, *SD* = 7.07) were recruited through publication board announcements and by a computerized sign-up system of the University of Aberdeen, UK. All participants were native English speakers and received course credits or 10 Pounds for their participation. Participants all signed a written consent form that was stored in a secure location in the Psychology Department. For this study approval was obtained from the Aberdeen School of Psychology Ethics committee.

### Design

The two interviewers (Interviewer 1 or 2) were fully counterbalanced over the experimental conditions. Participants were randomly assigned to one of three repeated interview conditions that differed as a function of delay (immediate & 2 days, N= 28, immediate & 7 days, N = 26 7 days & 9 days, N = 26).

### Procedure

A 2 minute videotape was shown to each individual participant on a high quality 17-inch computer screen. The participants were told to pay attention because they would be asked some questions about it later. The video depicts a story line about a woman walking home while being stalked by a man (See Appendix 1 for a description).

After the video, participants in the delayed interview condition (7 days & 9 days) were reminded about their upcoming appointments and asked to leave. The remaining participants all received their initial interview after completing an unrelated face recognition task that served as a 15-minute ‘filler.’ The face recognition task was considered not to interfere with the episodic recall task of the main experiment any more than would be the case for a regular eyewitness. In all immediate conditions, the first interview started after finishing the filler task. Each participant was then interviewed individually a second time by the same or different interviewer.

### Interviewers and interviews

#### Interviewers

Two female researchers were fully trained in aspects of the modified CI (see [,36]). Both female interviewers were approximately of the same age and had a similar background being graduates in Psychology with experience in conducting research. They were trained together. The two principle CI techniques that were used were to ask the witness to mentally reinstate the original context in which they had viewed the video and to report all the details that they could remember in full.

#### Training and Interviews

Each interviewer underwent a two-day training program in the modified CI which included background reading, lectures, role plays and constructive feedback in rapport building, context reinstatement, eliciting a detailed report, witness-centered questioning, use of extensive pauses, and not interrupting the witnesses. The interviewers were also asked to convey the ‘ground rules’ that witnesses should report as much detail as possible, but that they should not guess or make things up. If they did not know the answer to a question, witnesses were instructed that it was OK to say that they didn’t know. Both interviewers reached a similar level of proficiency having had their practice interviews checked several times and with extensive feedback on the quality during the training session.

In all conditions, Interview 1 and Interview 2 followed the same format. After rapport building, participants were asked to close their eyes and contextually reinstate the video before starting their free recall; this was followed by the specific questions. To make sure that the witness-centered questions were consistent across the subsequent interviewers, a list was designed containing one or two questions on 10 topics about scenes in the video. During the free recall, the interviewer numbered the topics to make sure that the follow-up questions would be asked in the same order of the recall of the participant. This list of topics was identical during both interviews.

### Scoring

Each interview was transcribed verbatim. This was followed by the removal of subjective statements (e.g., shady character), un-measurable information (e.g., the man in video was 1.80 meters tall) and utterances (e.g., maybe, I think) from the transcripts. The remaining information was divided in units of information. For example, if a participant described “the man was wearing a blue hoodie with a logo on the front” this was divided in six units “the man”, “was wearing”, “a blue”, “hoodie”, “logo”, “on the front”. Accuracy was scored by comparing every unit with the video recording.

Each unit of information at interview 1 and 2 was categorized in the following ways: consistent (same information given at Interview 1 and Interview 2), forgotten (information given at Interview 1 but not at Interview 2), and reminiscent (information given at Interview 2 but not at Interview 1). Consistent information was scored liberally, for instance, “the car kept moving” at Interview 1 and “the car didn’t stop” at Interview 2 was scored as consistent. Contradicting information across the interviews, like ‘a red jacket’ at Interview 1 and ‘a blue jacket’ during Interview 2, was also scored.

To calculate the inter-rater reliability between the two coders (who were not the interviewers) 20% of the transcripts were scored independently by both. The inter-rater reliability was calculated as the number of coding agreements divided by the total number of agreements and disagreements for each transcript [38]. The inter-rater reliability was 97%. The inter-rater agreement between the coders was also measured with Cohen’s Kappa, κ = 0.83.

## Results

To examine both the costs and benefits of repeated interviewing the data were analysed in terms of the amount of correct information recalled, as well as the numbers of the errors. Consistent, forgotten, reminiscence and contradicting information were analyzed in turn. Hypermnesia was measured as an increase in the total number of accurate details recalled across successive interviews. Reminiscence, was measured as the cumulative recall of new details across successive interviews, that is, the number of correct details from Interview 1 plus new details from Interview 2 following the analysis plan used in previous studies [9,]. Interviewer (same or different) was included as a between subject variable.

### Hypermnesia

To examine the effect of hypermnesia (an increase in the total amount of correct details reported in the repeated interview), a mixed-model analysis of variance (ANOVA) was performed with Repeated Interview (Interview 1 or 2) as within-subjects factor and Recall Delay (Immediate & 2 days, Immediate & 7 days, 7 days & 9 days), and Interviewer Identity (Same or Different) as between-subjects factors. Hypermnesia was observed across the repeated interviews with the number of correct details increasing from 63.35 (SD = 17.82) in Interview 1 to 66.03 (SD = 18.32) in Interview 2 (*F*(1, 74) = 6.00, *p* = .017, η^2^ = .07). No effect was found for Interviewer Identity (i.e., whether the interviewer was the same or different across interviews). However, there was an overall effect for the timing of the two interviews, *F*(2, 74) = 17.54, *p* < .001, η^2^ = .32, with the mean number of correct recalled details over both interviews for the conditions Immediate & 2 days, Immediate & 7 days, 7 Days & 9 days, being 68.20 (SD = 14.53), 74.86 (SD = 14.70), 51.02 (SD = 14.55) respectively (see Table 1). Post-hoc tests reveal that significantly fewer numbers of details were obtained when the repeated interviews occurred 7 days and 9 days after the event (Bonferroni, *p* < .05).

**Table 1:** The mean number of units reported during Interview 1 and 2 and Cumulative recall.

	First interview	Second interview	Cumulative recall*
Correct			
Immediate & 2 days	66.25 (14.17)	70.14 (17.06)	82.71 (17.69)
Immediate & 7 days	74.04 (15.80)	75.69 (16.51)	90.35 (18.62)
7 days & 9 days	49.77 (14.71)	52.27 (12.67)	60.85 (14.73)
Overall	63.35 (17.82)	66.03 (18.32)	77.97 (20.98)
Errors			
Immediate & 2 days	10.79 (4.54)	12.50 (5.25)	16.86 (6.94)
Immediate & 7 days	11.92 (6.93)	12.73 (6.36)	17.92 (8.54)
7 days & 9 days	13.46 (6.92)	14.38 (7.25)	19.50 (9.23)
Overall	12.06 (10.7)	13.20 (10.9)	18.09 (11.36)

Note. Standard deviations are in parentheses.

* Cumulative recall is the number of units from the first interview plus new details from the second interview.

### Reminiscence

To examine reminiscence, measured as the cumulative recall of new accurate details across the repeated interviews, a mixed-model ANOVA was performed with Repeated Interview (Interview 1 or 2) as within-subjects factor and Recall Delay (Immediate & 2 days, Immediate & 7 days, 7 Days & 9 days) and Interviewer (Same or Different) as between-subjects factor. The results confirmed the cumulative recall of new correct details across the repeated interviews increased from 63.35 (SD = 17.82) to 77.97 (SD = 20.98) in Interview 2 (*F*(1, 74) = 294.69, *p* < .001, η^2^ = .78). This was modified by an interaction between the repeated interviews and the delay between the two interviews, *F*(2, 74) = 4.23, *p* = .018, η^2^ = .02. Once again, no effect for interviewer identity was found. To further explore the interaction between these variables, correct recall was examined separately for Interview 1 and Interview 2 as a function of the delays between the interviews. The difference between initial recall during Interview 1 and cumulative correct recall was reduced when the interviews were held 7 & 9 days after the video (*F*(2, 74) = 20.53, *p* < .001, η^2^ = .35). In other words, the cumulative recall of correct details reported in both interviews did not differ when interviews were conducted immediately, but cumulative recall (reminiscence) was the lowest when the first interview occurred after a delay (see Table 1).

### Errors

To examine the effect of delay and repeated interviews on the errors, a mixed-model analysis of variance (ANOVA) was performed on both the total numbers of errors in Interview 1 and 2, and on the cumulative errors. Repeated Interview (Interview 1 or 2) served as a within-subjects factor and Delay (Immediate & 2 days, Immediate & 7 days, 7 Days & 9 days) and Interviewer (Same or Different) as between-subjects factor. There was an increase observed in the total numbers of errors across the repeated interviews from 12.06 (SD = 6.22) in Interview 1, to 13.20 (SD = 6.29) in Interview 2 (*F*(1, 74) = 8.66, *p* = .004, η^2^ = .10). There was also a significant interaction effect with Repeated Interview, Delay and Interviewer (*F*(2, 74) = 3.66, *p* = .030, η^2^ = .09). To explore this interaction effect three ANOVAs were conducted with Repeated Interview (Interview 1 or 2) as within-subjects factors and Interviewer Identity (Same of Different) as between-subjects factors at each recall delay (Immediate & 2 days, Immediate & 7 days, 7 days & 9 days). These additional ANOVAs revealed no significant interaction between Interview (Interview 1 or 2) and Interviewer Identity (Same of Different) when the interviews were conducted immediate and 2 days after the event (*F*(1, 26) = 1.40, *p* = .248, η^2^ = .05), after 7 and 9 days recall delay (*F*(1, 24) = 1.71, *p* = .204, η^2^ = .07) or in the immediate and 7 days recall delay condition (*F*(1, 24) = 4.11, *p* = .054, η^2^ = .15).

The cumulative number of new errors across the repeated interviews, also increased significantly from 12.06 (SD = 6.22) in Interview 1 to 18.09 (SD = 8.23) in Interview 2 (*F*(1, 74) = 249.40, *p* < .001, η^2^ = .76). The interaction between Repeated Interview, Delay and Interviewer just failed to reach significance (*F*(2, 74) = 2.72, *p* = .073, η^2^ = .10).

### Accuracy

A mixed-model analysis of variance (ANOVA) examined accuracy in each of the interviews as well as accuracy calculated from the cumulative recall data. Repeated Interview (Interview 1 or 2) served as within-subjects factor with Recall Delay (Immediate & 2 days, Immediate & 7 days, 7 days & 9 days) and Interviewer Identity (Same or Different) as between-subjects factors. The accuracy in the cumulative recall confirmed the increase of new errors across the repeated interviews, proportion accuracy decreased significantly from .84 (SD = .07) in Interview 1 to .81 (SD = .07) in Interview 2 (*F*(1, 74) = 93.77, *p* < .001, η^2^ = .53). There was also an effect of Delay on the accuracy of cumulative recall, *F*(2, 74) = 10.52, *p* < .001, η^2^ = .22. The proportion correct in cumulative recall for across the Immediate & 2 days, Immediate & 7 days, 7 Days & 9 days delays were .83 (SD = .06), .84 (SD = .06), .76 (SD = .06) respectively. Again, post-hoc tests indicated significant lower accuracy when the interviews occurred 7 days and 9 days after the event (Bonferroni, *p* < .05).

In addition, a significant interaction between Repeated Interviews, Delay and the Interviewer was found, *F*(2, 74) = 4.54, *p* = .014, η^2^ = .05. To explore this interaction three ANOVAs were conducted with Repeated Interview (Interview 1 or 2) as within-subjects factors and Interviewer Identity (Same of Different) as between-subjects factors at each recall delay (Immediate & 2 days, Immediate & 7 days, 7 days & 9 days). These additional ANOVAs revealed no significant interaction between Interview (Interview 1 or 2) and Interviewer Identity (Same of Different) when the interviews were conducted soon after the event (*F*(1, 26) = 0.28, *p* = .602, η^2^ = .01) or after a 7 days and 9 days recall delay, (*F*(1, 24) = 4.12, *p* = .054, η^2^ = .15). However, there did appear to be a significant interaction when interviews were conducted in the Immediate and 7 days recall delay condition (*F*(1, 24) = 4.82, *p* = .038, η^2^ = .17). The effect of the Interviewer Identity (Same or Different) on the cumulative accuracy was however, not straightforward and varies within every testing condition. We will comment on this further in the discussion.

The accuracy of recall did not differ between Interview 1 (*M* = .84, *SD* = .07) and Interview 2 (*M* = .83, *SD* = .07, *F*(1, 74) = 2.75, *p* = .102). There was however, an effect of Delay on accuracy, *F*(2, 74) = 10.40, *p* < .001, η^2^ = .21. Bonferroni post-hoc tests revealed that when Interview 1 and Interview 2 occurred 7 days and 9 days after the event, the reported details were significantly lower in accuracy compared to the two other recall delay conditions (Bonferroni, *p* < .05). Proportion correct recall for the delays Immediate & 2 days, Immediate & 7 days, 7 days & 9 days were .83 (SD = .05), .84 (SD = .06), .76 (SD = .08) respectively.

### Forgotten, consistent and reminisced details

Reminiscence appears to be a very common phenomenon in our data. A mean proportion of 0.21 of all information recalled during Interview 2 was new (see Table 2), although a proportion of 0.06 was incorrect information. The accuracy of the reminiscent items was 71% (SD = .14), which is in line with previous findings of Gilbert & Fisher [2] and Brock, Fisher, & Cutler [32]. Contradictory responses across the interviews were extremely rare, less than 0.01%. Although reminiscence and forgotten units of information were often correct (*M* = .68, *SD* = .14 and *M* = .71, *SD* = .16 respectively), consistent information appeared to be significantly more accurate (*M* = .88, *SD* = .06, *F*(2, 154) = 60.01, *p* < .001, η^2^ = .44). To test the assumption that consistency and accuracy are closely related, a Pearson correlation was calculated between accuracy in cumulative recall and the proportion of information that was recalled consistently across the interviews. The correlation appears to be low (*r* = .16, *p* = .121) indicating that consistency is not a reliable indicator of overall accuracy.

**Table 2:** Mean number of correct and incorrect forgotten, consistent and reminiscences information.

	Forgotten details		Consistent details		Reminiscent details	
Correct						
Immediate & 2 days	12.6 (4.6)	*.13*	53.7 (13.2)	*.54*	16.5 (10.2)	*.16*
Immediate & 7 days	14.7 (6.5)	*.14*	59.4 (13.0)	*.55*	16.3 (6.6)	*.15*
7 days & 9 days	8.6 (4.0)	*.11*	41.2 (12.3)	*.51*	11.1 (3.8)	*.14*
Overall	12.0 (8.9)	*.13*	51.4 (16.3)	*.53*	14.6 (12.7)	*.15*
Errors						
Immediate & 2 days	4.4 (2.9)	*.04*	6.4 (3.0)	*.07*	6.1 (3.6)	*.06*
Immediate & 7 days	5.2 (3.1)	*.05*	6.7 (4.6)	*.06*	6.0 (3.5)	*.05*
7 days & 9 days	5.1 (3.6)	*.06*	8.4 (5.1)	*.10*	6.0 (3.3)	*.08*
Overall	4.9 (5.6)	*.05*	7.2 (7.5)	*.08*	6.0 (6.0)	*.06*

Note. Distribution of the details in proportions are printed in italics, standard deviations are presented in parentheses.

To examine if Interviewer Identity across subsequent interviews had an effect on the social demands for being consistent, an ANOVA was calculated between the proportion consistent information of the total amount of information recalled by the participants and Interviewer (Same of Different). The results showed that the proportion of consistent information did not differ when the participants were interviewed by the same interviewer (*M* = .60, *SD* = .08) or by two different interviewers (*M* = .62, *SD* = .09, *F*(1, 79) = .55, p = .445).

## Discussion

This study was designed to examine the costs and benefits of repeated interviews and providing new knowledge which can be applied by practitioners. Eyewitnesses to a filmed event were interviewed twice using a Cognitive Interview procedure to examine the effects of variations in delay between the repeated interviews and the identity of the interviewer. The main findings were that across all conditions, a repeated interview yielded on average, 21% of previously unreported details with all participants reporting at least 1 item of new information. A novel finding is that the timing of the interviews had a significant effect on the amount of reminiscence. As predicted, participants who were interviewed immediately after the video provided more new details during their second interview than participants in the delay condition. Interestingly, this effect was also found when the two interviews were separated by an interval of 1 week. Hence, this research shows for the first time, how critical the timing of a first interview is and it supports the use of interview protocols where information can be gathered from witnesses as soon as possible after an event is witnessed [39]. While there was some forgetting over the repeated interviews, the total numbers of errors reported within an interview increased significantly with repeated interviewing. Importantly, the overall accuracy did not change across the repeated interviews. Accuracy of correct details reported decreased significantly, although not dramatically, from .84 in the first interview to .81 during the second interview.

The findings point to the practical conclusion that new, previously unreported details are recalled across high quality face-to-face Cognitive Interviews. This ‘reminiscence effect’ can be explained by conventional cognitive theory, which relates reminiscence to changes in retrieval cues from one interview to the next. These results show that cognitive interviewing does not ‘exhaust’ witness memory in an initial interview. Indeed because more information is retrieved in the initial interview (than would otherwise be recalled) there may be a greater chance that these details will be used as memory cues in future interviews and/or that an earlier cognitive interview reduces forgetting of details. Thus the timing of the first interview appears to be crucial in determining the strength of the effect of reminiscence and once again supports the importance of interviewing witnesses as soon as possible. In a forensic setting, an early interview using an appropriate interview protocol such as the Self-Administered Interview [39] may reduce forgetting. In addition, as a consequence of an early recall using the SAI, witnesses may recall new additional information during a following face-to-face interview. The benefits of using an SAI are currently being examined in real-life field settings with positive preliminary results [40].

In our data, consistency appears not to be strongly related with accuracy, a finding that is in line with the published literature [,,,]. In legal settings, inconsistency is assumed to be indicative of inaccuracy. Contradictory testimonies were extremely rare in our data. The use of research based protocols, trained interviewers, optimal encoding and test conditions may have contributed to the tendency for people to be consistent in their recall. Future studies should examine if the lack of a relationship between consistency and accuracy holds in less than optimal test conditions and over lengthier delays than the ones used here.

The identity of the interviewer appeared to have a significant but mixed effect on errors. This it is an intriguing finding because our two female interviewers underwent identical training and received feedback on their rapport building and interpersonal style to keep interview style and social dynamics to a minimum. The appearance of the interviewers in this study were quite similar, both Caucasian blond females of the same age. The interviewers were nevertheless easily distinguished as two different individuals, because they were of different nationalities (Dutch and Scottish). Other factors that might be important are whether or not the interviewers have similar status and are of the same gender but these are questions which can be pursued in future studies. The purpose of this study was to determine if a second interview with a new person elicited different information whilst minimising social confounding variable that might complicate the picture. Our mixed results, together with the findings of Bjorklund et al. [36] who reported higher errors rates when interviews were conducted by different interviewers, make clear that more research is needed in this area.

Many years of research have shown that the CI is a reliable and highly effective method for gathering eyewitness testimonies [16]. The novel finding of the current study is that two Cognitive Interviews can elicit more information than just one. Based on previous research outcome we expected that the CI would ‘exhaust’ initial recall and that nothing would be left for a second interview, however this was not the case. Following Gilbert & Fisher [2], who concluded that varying retrieval cues increases the amount of reminiscence, we anticipated an increase in previously unmentioned information across repeated interviews. The reminiscence effect might be even more appealing when varying retrieval cues are introduced in future research on repeated Cognitive Interviewing.

We found no accompanying drop in overall accuracy across the repeated interviews. Which is of critical importance, especially in applied settings [42]. As alluded to earlier, the interviews in this study were high quality interviews without any leading questions or suggestive information and the interviewers were well trained. The outcome may be different if repeated interviews involve suggestion and if the interviewees are vulnerable (see for example, 43). However, the current research indicates that an early CI conducted before a witness is asked misleading questions can reduce the likelihood of a witness responding based on false or misleading information [,44].

## Supporting Information

Appendix S1 **Description of the video.**
(DOCX)

## References

[1] Fisher RP, Brewer N, Mitchell G (2009) The relation between consistency and accuracy of eyewitness testimony: Legal versus cognitive explanations. In: Bull R Valentine T Williamson T Handbook of psychology of investigative interviewing: Current developments and future directions. John Wiley & Sons, Ltd. Retrieved onpublished at whilst December year 1111 from . doi:10.1002/9780470747599.ch8.

[2] Gilbert JA, Fisher RP (2006) The effects of varied retrieval cues on reminiscence in eyewitness memory. Appl Cogn Psychol 20: 723-739. doi:10.1002/acp.1232.

[3] Executive Scottish (2007) On the record: Evaluating the visual recording of joint investigative interviews with children. Edinburgh: Author.

[4] Odinot G, Wolters G, Lavender T (2008) Repeated partial eyewitness questioning causes confidence inflation but not retrieval-induced forgetting. Appl Cogn Psychol 23: 90-97. doi:10.1002/acp.1443.

[5] Shaw JS (1996) Increases in eyewitness confidence resulting from postevent questioning. J Exp Psychol Appl 2: 26-146. doi:10.1037/1076-898X.2.2.126.

[6] Shaw JS, Bjork RA, Handal A (1995) Retrieval-induced forgetting in an eyewitness-memory paradigm. Psychon Bull Rev 2: 249-253. doi:10.3758/BF03210965. Retrieved onpublished at whilst December year 1111 from 10.3758/BF03210965 24203660

[7] Chan JCK, Thomas AK, Bulevich JB (2009) Recalling a witnesses event increases eyewitness suggestibility. Psychol Sci 20: 66-73. doi:10.1111/j.1467-9280.2008.02245.x. PubMed: 19037905. Retrieved onpublished at whilst December year 1111 from 10.1111/j.1467-9280.2008.02245.x 19037905

[8] Davis D, Loftus EF (2007) Internal and external sources of misinformation in adult witness memory. In: Toglia MP Read JD Ross DF Lindsay RCL The handbook of psychology; Memory for events. Vol. 1 Mahwah, NJ: Erlbaum pp. 195-225.

[9] Bluck S, Levine LJ, Laulhere TM (1999) Autobiographical remembering and hypermnesia: A comparison of older and younger adults. Psych Younger Adults 14: 671-682. PubMed: 10632153. 10.1037//0882-7974.14.4.671 10632153

[10] Dunning D, Stern LB (1992) Examining the generality of eyewitness hypermnesia: A closer look at time delay and question type. Appl Cogn Psychol 6: 643-657. doi:10.1002/acp.2350060707.

[12] Hershkowitz I, Terner A (2007) The effect of repeated interviewing on children’s forensic statements of sexual abuse. Appl Cogn Psychol 21: 1131-1143. doi:10.1002/acp.1319.

[13] La Rooy D, Katz C, Malloy LC, Lamb ME (2010) Do we need to rethink guidance on repeated interviews? Psychol Public Policy Law 16: 373-392. doi:10.1037/a0019909.

[14] La Rooy D, Pipe ME, Murray JE (2005) Reminiscence and hypermnesia in children’s eyewitness memory. J Exp Child Psychol 90: 235-254. doi:10.1016/j.jecp.2004.11.002. PubMed: 15707861. 15707861

[15] Scrivner E, Safer MA (1988) Eyewitnesses show hypermnesia for details about a violent event. J Appl Psychol 73: 371-377. doi:10.1037/0021-9010.73.3.371. PubMed: 3182548. 3182548

[16] Memon A, Meissner CA, Fraser J (2010) The Cognitive Interview: A meta-analytic review and study space analysis of the past 25 years. Psychol Public Policy Law 16: 340-372. doi:10.1037/a0020518.

[17] La Rooy D, Lamb ME, Pipe ME (2008) Repeated interviewing: A critical evaluation of the risks and potential benefits. In: Kuehnle K Connell M. Child sexual abuse: Research, evaluation, and testimony for the courts. New Yersey. John Wiley & Sons Ltd. pp 327-364.

[18] Brewer N, Potter R, Fisher RP, Bond N, Luszcz MA (1999) Beliefs and data on the relationship between consistency and accuracy of eyewitness testimony. Appl Cogn Psychol 13: 297-313. doi:10.1002/(SICI)1099-0720(199908)13:4<297::AID-ACP578>3.0.CO;2-S.

[19] La Rooy D, Dando C (2010) Witness Interviewing. In: Towl G Crighton D Forensic Psychology. Blackwell Publishing Ltd. pp. 195-206.

[20] Fisher RP, Geiselman ER (1992) Memory-enhancing techniques for investigative interviewing. The cognitive interview. Springfield, IL: Charles C. Thomas.

[21] Brock P, Fisher RP, Cutler BL (1999) Examining the cognitive interview in a double-test paradigm. Psychol Crime Law 5: 29-45. doi:10.1080/10683169908414992.

[22] McCauley MR, Fisher RP (1995) Facilitating children’s eyewitness recall with the revised cognitive interview. J Appl Psychol 80: 510-516. doi:10.1037/0021-9010.80.4.510. PubMed: 7642461. 7642461

[23] Memon A, Wark L, Bull R, Kohnken G (1997) Isolating the effects of the cognitive interview and structured interview. Br J Psychol 88: 179-198. doi:10.1111/j.2044-8295.1997.tb02629.x.

[24] Read JD, Connolly DA (2007) The effects of delay on long-term memory for witnessed events. In: Toglia MP Read JD Ross DF Lindsay RCL Handbook of Psychology. Vol. 1 Mahwah, NJ: Erlbaum pp. 117-155.

[25] Erdelyi MH (1996) The recovery of unconscious memories: Hypermnesia and reminiscence. Chicago: University of Chicago Press.

[26] Payne DG (1987) Hypermnesia and reminiscence in recall: A historical and empirical review. Psychol Bull 101: 5-27. doi:10.1037/0033-2909.101.1.5.

[27] Ebbinghaus H (1964/1885) Memory: A Contribution to Experimental Psychology. New York. 10.5214/ans.0972.7531.200408 PMC4117135 25206041

[28] Wixted JT, Ebbesen EB (1991) On the form of forgetting. Psychol Sci 2: 409-415. doi:10.1111/j.1467-9280.1991.tb00175.x.

[29] Wixted JT, Ebbesen EB (1997) Genuine power curves in forgetting: A quantitative analysis of individual subject forgetting functions. Mem Cogn 25: 731-739. doi:10.3758/BF03211316. PubMed: 9337591. 9337591

[30] Bjork RA (1988) Retrieval practice and the maintenance of knowledge. In: Gruneberg MM Morris PE Skyes RN Practical aspects of memoryII. New York: Wiley pp. 396-401.

[31] Odinot G, Wolters G (2006) Repeated recall, retention interval and the accuracy-confidence relation in eyewitness memory. Appl Cogn Psychol 20: 973-985. doi:10.1002/acp.1263.

[32] Fisher RP, Cutler BL (1995) The relation between consistency and accuracy of eyewitness testimony. In: Davies G Lloyd Bostock S McMurran M Wilson C Psychology, law and criminal justice: International developments in research and practice. Berlin: de Gruyter pp. 21-28.

[33] McNally RJ (2003) Remembering trauma. Cambridge, MA: The Belknap Press of Harvard University Press.

[34] Talarico JM, Rubin DC (2003) Confidence, not consistency, characterizes flashbulb memories. Psychol Sci 14: 455-461. doi:10.1111/1467-9280.02453. PubMed: 12930476. 12930476

[35] Odinot G, Wolters G, van Giezen A (2012) Repeated suggestive questioning, consistency and the accuracy-confidence relation in eyewitness event memory. Psychol Crime Law, 18: 27–47. doi:10.1080/1068316X.2012.660152. PubMed: 22523466.22523466

[36] Bjorklund DF, Cassel WS, Bjorklund BR, Douglas Brown R, Park CL, Ernst K, Owen FA (2000) Social demand characteristics in children’s and adults’ eyewitness memory and suggestibility: The effect of different interviewers on free recall and recognition. Appl Cogn Psychol 14: 421-433. doi:10.1002/1099-0720(200009)14:5<421::AID-ACP659>3.0.CO;2-4.

[37] Memon A (2006) The cognitive interview. In: Hargie O A handbook of communication skills (3rd ed.). London and New York: Routledge.

[38] Tinsley HEA, Weiss DJ (2000) Interrater reliability and agreement. In: Tinsley HEA Brown SD Handbook of applied multivariate statistics and mathematical modeling. San Diego: Academic Press pp. 95-124. Retrieved onpublished at whilst December year 1111 from 10.1016/B978-012691360-6/50005-7

[39] Gabbert F, Hope L, Fisher RP (2009) Protecting eyewitness evidence: Examining the efficacy of a self-administered interview tool. Law Hum Behav 33: 298-307. doi:10.1007/s10979-008-9146-8. PubMed: 18561007. 18561007

[40] Gabbert F (2010) Field trials of the Self-Administered Interview (SAI) recall tool with real eyewitnesses. Presentation at the annual meeting of the European Association of Psychology and Law, Gothenburg, Sweden 15-18 June.

[41] Smeets T, Candel I, Merckelbach H (2004) Accuracy, completeness, and consistency of emotional memories. Am J Psychol 117: 595-609. doi:10.2307/4148994. PubMed: 15605961. 15605961

[42] Fisher RP (1995) Interviewing victims and witnesses of crime. Psychol Public Policy Law 1: 732-764. doi:10.1037/1076-8971.1.4.732.

[43] Ceci SJ, Crotteau ML, Smith E, Loftus EF (1994) Repeatedly thinking about non-events: Source misattributions among preschoolers. Conscious Cogn 3: 388-407. doi:10.1006/ccog.1994.1022.

[44] Gabbert F, Hope L, Fisher RP, Jamieson K (2012) Protecting against misleading post-event information with a Self-Administered Interview. Appl Cogn Psychol 26: 568-575. doi:10.1002/acp.2828.

[45] Memon A, Zaragoza M, Clifford BR, Kidd L (2010) Inoculation or antidote? The effects of Cognitive Interview timing on false memory for forcibly fabricated events. Law Hum Behav 34: 105-117. doi:10.1007/s10979-008-9172-6. PubMed: 19301110. 19301110

