# Supplementary material for: Are Two Interviews Better Than One? Eyewitness Memory across Repeated Cognitive Interviews
Source: PLoS One. 2013 Oct 3;8(10):e76305. doi: 10.1371/journal.pone.0076305 (PMC3789709; doi:10.1371/journal.pone.0076305)
Supplement: Appendix S1 — Description of the video. (DOCX) [file pone.0076305.s001.docx]

**Supporting information S1**

*Description of the video S1*

In the video, a man follows a woman while she is walking to her house. They cross a street and after she enters the house, the man approaches the front door. He fiddles with the lock of the door and, through the glass panel in the door, the viewer sees that he is holding a hammer in his hand. The video then shows the woman fully dressed, sleeping on a sofa bed in an untidy bedroom. The man opens the front door, enters the hallway and walks slowly around the house looking in various rooms. He moves from room to room until he finds the woman in the bedroom. When he sees her, he looks at his hammer and closes the door. The video shows the closed door while we hear a loud female scream. Then, the man opens the bedroom door, runs through the hallway and leaves the house through the front door. The video ends.
